# The Nature of Patient- and Family-Centred Care for Young Adults Living with Chronic Disease and their Family Members: A Systematic Review

**DOI:** 10.5334/ijic.3110

**Published:** 2018-05-18

**Authors:** David Allen, Nerina Scarinci, Louise Hickson

**Affiliations:** 1 The HEARing CRC, The University of Queensland, AU; 2 The HEARing CRC, Department of Audiology and Speech Pathology, The University of Melbourne, 550 Swanston Street, Carlton, Victoria, AU; 3 School of Health and Rehabilitation Sciences, The University of Queensland, AU

**Keywords:** Patient- and Family-Centred Care, Young Adults, Chronic Disease

## Abstract

**Background and Aim:**

The published literature addressing the nature of patient- and family-centred care (PFCC) among young adults (16–25 years old) living with chronic disease and their family members is diverse. The aim of this systematic review was to collect and interpretatively synthesise this literature to generate a conceptual understanding of PFCC in this age group.

**Method:**

From an initial pool of 10,615 papers, 51 were systematically identified as relevant to the research question and appraised using the Critical Appraisal Skills Programme tools. A total of 24 papers passed the quality appraisal and proceeded to a qualitative meta-synthesis.

**Results:**

The qualitative meta-synthesis revealed three major elements of PFCC relevant to young adults living with chronic disease and their family members: (1) patients and practitioners felt able to engage with each other on an emotional and social level; (2) patients and families felt empowered to be part of the care process; and (3) patients and families experienced care as effective at addressing their individual needs.

**Conclusion:**

There is agreement among young adult patients and families about what constitutes PFCC in a chronic disease setting, independent of the aetiology of the pathological process. Patients and families also have strong feelings about how practitioners can achieve PFCC in practice. These findings have implications for the delivery of health services to young adults living with chronic disease and their family members.

## Introduction

People living with chronic health conditions, defined as diseases that persist over a long time course, that are recurrent, and where the focus is on symptom management rather than curing the underlying disease process [1], may require multiple health care providers to meet their needs, requiring significant interaction between patients and professionals [2]. One approach to improving informational transfer and therefore the integration of health care is patient-centred care [3], in which the patient is the centre of care interactions, the primary decision-maker, and a primary source of information. Patient-centredness has been recognised as a core principle underlying the integration of health systems [4].

When treating younger people and children, the patient-centred approach is often extended to “Patient- and Family-Centred Care” (PFCC), a term that emphasises the patient as being able to participate in their care, but gaining significant support from their family [5]. PFCC can be a powerful way of improving communication between the patient and family and the health care team, making the patient and their family more equal and active participants in the health care team [6].

The dominant models of patient-centred care have traditionally been developed in general practice [3,6]. As a result, these models have focused on the patient populations who attend general practices, predominately adults over the age of 25 [7]. Similarly, the development of models of family-centred care has primarily been in the context of younger children attending health services with their parents [8]. Patients between adolescence and adulthood have not been regularly engaged in research determining the nature of PFCC.

Arnett [9] describes the time between adolescence and young adulthood, which he terms “emerging adulthood”, as marked by changes in demographics, self-identity, and ideology [10]. Demographically, emerging adulthood is a time of transition in which young adults may embark on tertiary or vocational study, begin their working lives, and move away from their caregivers – potentially to form romantic relationships or families of their own [11]. It is also marked by the development of self-identity as an individual, separate from family. In a health care context, emerging adults claim from their parents and caregivers the rights to hold their health care information, and to use it to make decisions about their lives and bodies [12]. These sorts of changes, particularly changes in the composition of the family unit, have important implications for the design and implementation of PFCC.

## Background and Significance

It has been shown that PFCC in chronic disease care requires practitioners to take a role in legitimising and validating the patient’s experience of illness, encouraging hope for the future, and advocating for their rights on an ongoing basis [13]. As these aspects of PFCC are driven by the continuing nature of the chronic condition and the requirement for the patient to manage their health on an ongoing basis, they are not universally present in acute care. As such, investigation in chronic care specifically is necessary to bring forward these factors.

It is currently unclear what young adults living with chronic diseases and their family members identify as PFCC, and the research on this topic is diverse. The aim of this systematic review is to interpretatively synthesise reports of studies addressing the nature of PFCC among emerging adults living with chronic disease and their family members to generate an understanding of PFCC that addresses the needs of them and their families. The research question for the review was “What is the nature of PFCC as defined by young adults living with chronic disease and their family members?”

## Method

### Data Sources

A range of search strategies were used to identify literature for consideration for this review. The search strategies for this systematic review are presented in Table 1. CINAHL Terms and MeSH Headings relevant to PFCC, emerging adults, and adolescents were used for initial searches in CINAHL Complete and MEDLINE. From this, additional keywords were identified, which were incorporated into search strategies for MEDLINE (via EBSCOHost), CINAHL Complete (via EBSCOHost), PsycINFO and EMBASE in consultation with a librarian specialising in health sciences. Due to the discussion of PFCC over a long period of time in the psychological literature, no date restriction was placed on the searches.

**Table 1:** Search strategies used to identify papers.

Database	Search Date	Search Term	Total	Retained

MEDLINE	15/09/15	(MH Adolescent OR MH Young Adult OR MH Transition to Adult Care) NOT (MH Aged OR MH Middle Aged) AND (MH Patient-Centered Care OR MH Professional-Family Relations OR MH Personal Autonomy OR MH Patient Participation OR MH Professional-Patient Relations)	4802	187
EMBASE	21/09/15	(‘adolescent’/exp OR ‘young adult’/exp OR ‘transition to adult care’/exp) NOT (‘aged’/exp OR ‘middle aged’/exp) AND (‘holistic care’/exp OR ‘patient decision making’/exp OR ‘patient autonomy’/exp OR ‘personal autonomy’/exp OR ‘family centered care’/exp OR ‘patient participation’/exp OR ‘doctor patient relation’/exp) AND [embase]/lim	1853	213
CINAHL	22/09/15	((MH “Adolescence”) OR (MH “Young Adult”)) NOT ((MH “Aged”) OR (MH “Middle Age”)) AND ((MH “Professional-Patient Relations”) OR (MH “Physician-Patient Relations”) OR (MH “Patient Centered Care”) OR (MH “Professional-Family Relations”) OR (MH “Family Centered Care”) OR (MH “Patient Autonomy”) OR (MH “Decision Making, Patient”))	3025	210
PsycINFO	28/09/15	Index Terms: “client centered therapy” OR Index Terms: “client participation” OR Index Terms: “self determination” OR FirstPage: “patient-centered” OR FirstPage: “patient-centred” OR FirstPage: “patient centered” OR FirstPage: “patient centred” OR FirstPage: “person-centered” OR FirstPage: “person centered” OR FirstPage: “person centred” OR FirstPage: “person-centred” OR FirstPage: “family-centred” OR FirstPage: “family centred” OR FirstPage: “family-centered” OR FirstPage: “family centered” OR FirstPage: “physician-patient” OR FirstPage: “physician-family” OR FirstPage: “practitioner-patient” OR FirstPage: “practitioner-family” OR FirstPage: “clinician-patient” OR FirstPage: “clinician-family” OR FirstPage: “shared decision making” AND Age Group: Adolescence (13 to 17 yrs) OR Young Adulthood (18 to 29 yrs) AND NOT Age Group: Neonatal (birth to 1 mo) OR Infancy (2 to 23 mo) OR Preschool Age (2 to 5 yrs) OR Middle Age (40 to 64 yrs) OR Aged (60 yrs & older) OR Very Old (85 yrs & older)	935	56
Articles identified by hand			50	50
Total			10665	716

### Study Selection

**Figure 1:**
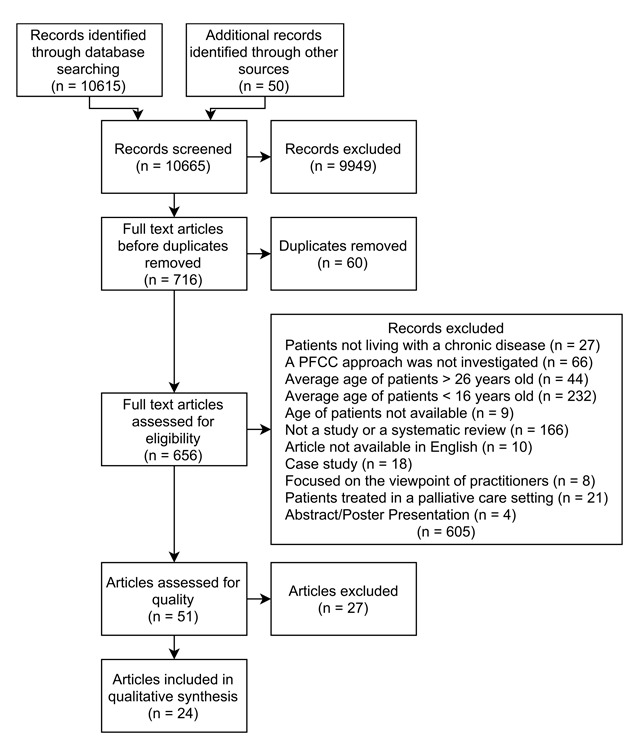
Flow of articles through appraisal process.

The movement of reports through the search, selection, and quality appraisal processes can be seen in Figure 1.

#### Inclusion Criteria

- Patients were living with a chronic disease
- The nature of a PFCC approach was investigated
- Average age of patients < 26 years old
- Average age of patients ≥ 16 years old
- The perspective of either patients or their family members were investigated, or a systematic review of such studies was conducted
- Article was available in English
- At least three participants were involved
- The study did not focus exclusively on the viewpoint of practitioners
- Patients were not being treated in a palliative care setting

A total of 51 papers passed this screening process and proceeded to the quality appraisal stage, including 46 reports of qualitative studies, two reports of quantitative studies, and three systematic reviews of published literature. In order to ensure that the inclusion criteria were applied appropriately, of the 656 papers identified for full-text checking, 10% (n = 66) were randomly selected, and independently assessed against the eligibility criteria by the second and third authors for agreement on their inclusion or exclusion. There was disagreement on two papers, which were then discussed and consensus was reached. The initial rating of the first author was agreed to in both cases.

### Quality Appraisal

The Critical Appraisal Skills Programme (CASP) checklists [14,15,16,17,18] were chosen as a screening tool as they have been used in a variety of existing systematic reviews, and do not rely on extensive theoretical knowledge of qualitative research [19]. After a review of systematic reviews using the CASP checklists, it was decided that papers would have to score a “Yes” on every relevant question of the appropriate CASP checklist to proceed to data extraction. After piloting the Randomised Controlled Trial [16], Case Control [17], and Cohort [18] CASP checklists, the Randomised Controlled Trial checklist was found to be too restrictive, and so the Cohort and Case Control checklists were combined, and an ethics criterion added, to form a Quantitative Research Study checklist. The modified tools are listed in Table 2.

**Table 2:** Modified CASP Tools used for quality appraisal.

**Qualitative Research Study**		**Qualitative Systematic Review**	

1.	Was there a clear statement of the aims of the research?	1.	Did the review address a clearly focused question?
2.	Is a qualitative methodology appropriate?	2.	Did the authors look for the right type of papers?
3.	Was the research design appropriate to address the aims of the research?	3.	Do you think all the important, relevant studies were included?
4.	Was the recruitment strategy appropriate to the aims of the research?	4.	Did the review’s authors do enough to assess the quality of the included studies?
5.	Was the data collected in a way that addressed the research issue?	5.	If the results of the review have been combined, was it reasonable to do so?
6.	Has the relationship between researcher and participants been adequately considered?		
7.	Have ethical issues been taken into consideration?		
8.	Was the data analysis sufficiently rigorous?		
9.	Is there a clear statement of findings?		
**Quantitative Research Study**			

1.	Did the study address a clearly focused issue?		
2.	Did the authors use an appropriate method to answer their question?		
3.	Were the cases recruited in an acceptable way?		
4.	Were the controls selected in an acceptable way?		
5.	Was the exposure accurately measured to minimise bias?		
6.	Was the outcome accurately measured to minimise bias?		
7.	Have the authors taken account of the potential confounding factors in the design or in their analysis?		
8.	Was the follow up of subjects complete enough? Was the follow up on subjects long enough?		
9.	Have ethical issues been taken into consideration?		
10.	Do you believe the results?		

The results of the application of the CASP tools for the 51 papers that passed to the quality appraisal stage can be seen in Tables 3, 4 and 5. In each table, “Y” indicates a positive answer to the relevant question, “N” indicates a negative answer, and “?” indicates that it was unclear whether the response should be positive or negative. Further clarification of unclear responses was not required, as each of these papers had already been excluded by a clear negative answer elsewhere in the tool.

**Table 3:** Quality appraisal results for assessed systematic reviews.

Systematic Review	Q1	Q2	Q3	Q4	Q5	Included

Anastasiadou, Medina-Pradas [23]	Y	Y	Y	N	N	N
Fegran, Hall [24]	Y	Y	Y	Y	Y	Y
Hussen, Chahroudi [25]	N	Y	?	N	Y	N

“Y” = Yes, “N” = No, “?” = question was unable to be answered clearly in this case.

**Table 4:** Quality appraisal results for assessed quantitative papers.

Quantitative Report	Q1	Q2	Q3	Q4	Q5	Q6	Q7	Q8	Q9	Q10	Included

Mauerhofer, Bertchold [26]	Y	Y	Y	Y	N	N	N	Y	Y	Y	N
Sonneveld, Strating [27]	Y	Y	Y	N	Y	Y	N	N	Y	Y	N

“Y” = Yes, “N” = No, “?” = question was unable to be answered clearly in this case.

**Table 5:** Quality appraisal results for assessed qualitative papers.

Qualitative Report	Q1	Q2	Q3	Q4	Q5	Q6	Q7	Q8	Q9	Included

Brumfield and Lansbury [28]	Y	Y	Y	Y	Y	N	Y	Y	Y	N
Cochrane, Sharpe [29]	Y	Y	Y	?	?	N	?	?	Y	N
Darrah, Magil-Evans [30]	Y	Y	Y	Y	Y	Y	Y	Y	Y	Y
Davis-Brown, Carter [31]	Y	Y	N	N	N	N	N	N	N	N
Delman, Clark [32]	Y	Y	Y	Y	Y	Y	Y	Y	Y	Y
Dogba, Rauch [33]	Y	Y	Y	Y	Y	Y	Y	?	N	N
Doig, Fleming [34]	Y	Y	Y	Y	Y	Y	Y	Y	Y	Y
Dovey-Pearce, Hurrell [35]	Y	Y	Y	Y	Y	Y	Y	Y	Y	Y
Dunsmore and Quine [36]	Y	Y	Y	Y	Y	Y	N	Y	N	N
Dupuis, Duhamel [37]	Y	Y	Y	Y	Y	N	Y	Y	Y	N
Fair, Sullivan [38]	Y	Y	Y	Y	Y	N	Y	?	Y	N
Garvie, Lawford [39]	Y	Y	Y	Y	Y	Y	Y	Y	Y	Y
Gerten and Hensley [40]	N	Y	N	N	N	Y	Y	Y	Y	N
Gillard and Roark [41]	Y	Y	Y	Y	N	Y	Y	N	N	N
Gilmer, Ojeda [42]	Y	Y	Y	Y	Y	Y	Y	Y	Y	Y
Grealish, Tai [43]	Y	Y	Y	Y	Y	Y	Y	Y	Y	Y
Harper, Dickson [44]	Y	Y	Y	Y	Y	Y	Y	Y	Y	Y
Hauser and Dorn [45]	Y	Y	Y	Y	Y	?	Y	N	Y	N
Honey, Boughtwood [46]	Y	Y	N	Y	N	?	?	Y	Y	N
Larivière-Bastien, Bell [47]	N	Y	N	N	Y	N	Y	Y	Y	N
Ledford [48]	Y	Y	Y	N	Y	N	Y	Y	Y	N
Lee, Munson [49]	Y	Y	Y	Y	Y	Y	Y	Y	Y	Y
Lester, Marshall [50]	Y	Y	Y	Y	Y	N	Y	Y	Y	N
Lewis and Noyes [51]	Y	Y	N	Y	N	Y	Y	Y	Y	N
Lucksted, Essock [52]	Y	Y	Y	Y	Y	Y	Y	Y	Y	Y
Miles, Edwards [53]	Y	Y	Y	Y	Y	N	Y	N	Y	N
Munson, Jaccard [54]	Y	Y	Y	Y	Y	Y	Y	Y	Y	Y
Nilson, Schachter [55]	Y	Y	Y	Y	Y	Y	Y	Y	Y	Y
Offord, Turner [21]	Y	Y	Y	Y	Y	Y	Y	Y	Y	Y
Olsen and Sutton [56]	N	Y	N	N	Y	N	Y	Y	Y	N
Parron [57]	Y	Y	?	?	?	?	?	N	Y	N
Patterson and Lanier [58]	Y	Y	Y	Y	Y	Y	Y	Y	Y	Y
Price, Corbett [59]	Y	Y	Y	Y	Y	N	Y	Y	Y	N
Racine, Lariviere-Bastien [60]	Y	Y	Y	N	Y	N	N	Y	Y	N
Reiss, Gibson [61]	Y	Y	Y	Y	Y	Y	Y	Y	N	N
Rudgley [62]	Y	Y	Y	Y	Y	Y	Y	Y	Y	Y
Rydström, Ygge [63]	Y	Y	Y	Y	Y	Y	Y	Y	Y	Y
Saaltink, Mackinnon [64]	Y	Y	Y	Y	Y	Y	Y	Y	Y	Y
Sasse, Aroni [65]	Y	Y	Y	Y	Y	Y	Y	Y	Y	Y
Sawin, Rauen [66]	N	Y	N	N	Y	N	Y	Y	Y	N
Shaw, Southwood [67]	Y	Y	Y	Y	Y	Y	Y	Y	Y	Y
Sly, Morgan [22]	Y	Y	Y	Y	Y	Y	Y	Y	Y	Y
Stewart, Law [68]	Y	Y	Y	Y	Y	Y	Y	Y	Y	Y
Swift, Hall [69]	Y	Y	Y	Y	Y	Y	Y	Y	Y	Y
van Staa, Jedeloo [70]	Y	Y	Y	Y	Y	Y	Y	Y	N	N
Webster and Harrison [71]	Y	Y	Y	Y	Y	Y	Y	Y	Y	Y

“Y” = Yes, “N” = No, “?” = question was unable to be answered clearly in this case.

Of three systematic reviews identified in the search, two were excluded as neither had identified a quality appraisal methodology. Of 46 qualitative studies, 23 were excluded. The most common reason for exclusion was insufficient consideration of the relationship between researcher and participants (n = 13), either by not situating the researcher in the research or by not taking steps to reduce the researcher’s effect on the results, such as independent coding of data by more than one researcher or review of coding by others on the research team. Several papers were also excluded due to a failure to report findings using the voices of the participants (n = 6), or for not collecting the data in an appropriate way (n = 5). Two quantitative reports were both excluded after quality appraisal due to insufficient consideration of potential confounding factors. The 24 remaining papers, which were all reports of qualitative papers or systematic reviews of qualitative papers, proceeded to synthesis.

Of the 51 papers that proceeded to quality appraisal, 12 (24%) were randomly selected, and these were independently assessed by the second and third authors according to the quality appraisal tools. There was disagreement on two papers, which were then discussed until consensus was reached. In one of these cases the first author’s decision was agreed to, and in the other case the paper was subsequently removed from the review.

### Data Extraction and Synthesis

Initially, each of the 24 full papers was read by the first author, and general details of each paper were recorded, including the number of participants, their relationship to the patients, the patients’ diagnoses, the data collection method and analysis style, and a broad outline of the findings. These may be seen in Table 6.

**Table 6:** General details of included papers.

Paper	Year	Aetiology	Included Groups	Participants	Patient age mean [range] (sd)	Phenomenon of interest	Method of Data Collection	Method of Data Analysis	Primary Findings

Darrah, Magil-Evans [30]	2002	Cerebral Palsy	Emerging Adults, Parents	38 families	[19–23]	Satisfaction with care	Questionnaire, Interview	Content Analysis	Caring and Supportive People Fighting and Fatigue Communication and Information Disability Awareness
Delman, Clark [32]	2015	Serious Mental Illness	Emerging Adults	24 patients	24 [19–30]	Facilitators and barriers to shared decision-making	Interview	Inductive Thematic Analysis	Facilitators: psychiatrist’s interest in the patient’s perspective support of other mental health providers personal growth self-confidence greater availability of the psychiatrist
									Barriers: short duration of meetings Psychiatrist’s resistance to the patient’s perspective limited self-efficacy
Doig, Fleming [34]	2009	Traumatic Brain Injury	Emerging Adults, Parents	12 patients and parents. Three therapists also interviewed.	24.7 (6.9)	Experience of a goal-directed therapy programme	Interview	Framework method	Provision of Structure Goals and Motivation Goal ownership Impact of awareness on participation Challenges Family Involvement Satisfaction and Progress Cognitive Function Goal Evolution Priorities
Dovey-Pearce, Hurrell [35]	2005	Diabetes	Emerging Adults	Interviews: 19 patients Focus Groups: 8 patients	Interviews: 19.9 (3.12) Focus groups: 19.4 (2.67)	Suggestions for appropriate diabetes service	Interview; Focus Group	Framework Approach	Diagnosis Continuity of staff contact Influence of age on care Interactions with staff Access and Environment Suggestions for service development
Fegran, Hall [24]	2014	Various	Emerging Adults	18 studies – metasynthesis		Adolescents’ and young adults’ transition experiences	Literature search	Qualitative Metasynthesis	Facing changes in significant relationships Moving from a familiar to an unknown ward culture Being prepared for transfer Achieving responsibility
Garvie, Lawford [39]	2009	HIV-1	Emerging Adults	17 patients	19.93 (1.29) [17.6–22.5]	Suggestions for appropriate Modified Directly Observed Therapy (MDOT) adherence intervention.	Focus Group	Content Analysis	Barriers to adherence MDOT Provider characteristics Location and safety of MDOT interactions Communication between MDOT provider and participant Logistics of MDOT interactions Duration of MDOT intervention Additional services to be provided during MDOT interaction Feasibility and acceptance of MDOT program Potential barriers to MDOT program
Gilmer, Ojeda [42]	2012	Mental Health Disorders	Emerging Adults, Parents	75 patients,14 parents	[18–24]	Needs for Mental Health and other services	Focus Group	Inductive Thematic Analysis	Mental health and substance abuse services Services that foster a transition to independence
Grealish, Tai [43]	2013	Psychosis	Emerging Adults	9 patients	16.4 [14–18]	Empowerment from the perspective of young people with psychosis	Interview	Interpretative Phenomenological Analysis	Individual control and choice vs inflexibility Being listened to, respected, and validated Communication Response of services Coping and structure Quality of relationship and support
Harper, Dickson [44]	2014	Mental Health Disorders	Emerging Adults	10 patients	[16–18]	Experiences of 16–18 year old Mental Health Service users	Interview	Interpretative Phenomenological Analysis	Developmentally attuned services Power differentials Parental involvement Developing self-expression Continuity and loss of relationships
Lee, Munson [49]	2006	Mental Health Disorders	Emerging Adults	389 patients	17 [17–17]	Attitudes towards mental health services among young adults in foster care	Interview	Thematic Analysis	Positive experiences are associated with beneficial care and relationships with a mental health professional; negative experiences were associated with concerns about treatment, poor relationships with a mental health professional, and unprofessional practice.
Lucksted, Essock [52]	2015	Psychosis	Emerging Adults	32 patients	23 [<20–34]	Views of engagement in an early intervention program for psychosis	Interview	Thematic Analysis	Individualised care Focus on life goals Effectiveness Warm respect
									Program attributes Team structure Setting and location Medication management approach Active outreach
									Family member influences Promoting engagement Deterring engagement Personal attributes
Munson, Jaccard [54]	2012	Mood Disorders	Emerging Adults	60 patients	20.97 (2.08)	Experiences of mental health service use during the transition to adulthood	Interview	Grounded Theoretic Analysis	Dynamic nature of service utilisation over time Core factors that impact service use at any given time
Nilson, Schachter [55]	2012	Haemophilia	Emerging Adults	18 patients	25.2 [17–31]	Health care-related knowledge, attitudes, and behaviours of young men with haemophilia	Interview (face to face and by telephone)	Constant Comparative Method	Reluctance to acknowledge having mild haemophilia Experiential learning trumps advice from the haemophilia team Negative reception to the health care team’s approaches Strategies for managing potential bleeds: watch and wait
Offord, Turner [21]	2006	Anorexia Nervosa	Emerging adults	7 patients	[16–23]	Experiences of treatment and discharge of young adults in inpatient treatment for anorexia nervosa	Interview	Interpretative Phenomenological Analysis	Removal from Normality vs. connecting with the outside world Suspension of real life Normality around mealtimes Suspension in development, compounding a sense of isolation Contrasts in structure and support
									Treated as another anorexic vs. a unique individual in distress Staff assumptions about eating disorders Standardised programmes Physical recovery prioritised over psychological recovery Recognising the eating disorder as a symptom A genuinely holistic approach
									Control and collaboration Initial taking away of control A structured containment Powerlessness, punishment and inadequacy Doing battle Collaborating in one’s own care Collaborating within therapy Preparing for discharge – handing back control
									The importance of peer relationships Distance from peers in the outside world Being alongside peers in distress – acceptance versus segregation Being alongside peers with anorexia nervosa – identification versus competition
Patterson and Lanier [58]	1999	Special Health Care Needs (Chronic illnesses or physical disabilities)	Emerging Adults	7 patients	24.3 (6.47) [17–33]	Experiences of, and facilitators and barriers to transition from paediatric care to adult care.	Focus Group	Grounded Theoretic Analysis	
Rudgley [62]	2013	Attention Deficit Hyperactivity Disorder	Emerging Adults	4 patients	[18–19]	Experiences of transition from paediatric to adult care of young adults with ADHD	Interview	Interpretative Phenomenological Analysis	Personal experience of ADHD diagnosis and treatment Impact of ADHD on self and relationships Living with ADHD Moving on
Rydström, Ygge [63]	2013	HIV	Emerging Adults	10 patients	18 [15–21]	Experiences of young people growing up with innate or early acquired HIV infection	Interview	Content Analysis	To protect oneself from the risk of being stigmatised To be in control Losses in life, but HIV is not a big deal Health care/health care providers Belief in the future
Saaltink, Mackinnon [64]	2012	Intellectual Disabilities	Emerging Adults, Parents, Siblings	4 patients,4 mothers,2 siblings	[14–18]	The negotiation of the right to participate in shared decision making in a family context	Interview	Interpretative Phenomenological Analysis	Autonomous participation Participation and protection: guidance and parents’ choice Decision-making processes as normal and natural Enabled choices
Sasse, Aroni [65]	2013	Various chronic issues, particularly eating disorders	Parents	17 parents	16 (1.4) [13–18]	Parental perspectives on confidential consultations between their adolescent children and health care providers	Interview	Content and Thematic Analysis	Variation in parental views about confidential consultations for adolescents The role of a parent: essential to their child expert on their child legal guardian of their child
									The influence of trust
Shaw, Southwood [67]	2004	Juvenile Idiopathic Arthritis	Emerging Adults, Parents	12 adolescents, 14 parents of adolescents, 18 young adults, 9 parents of young adults.	Adolescents: 16 [13–18] Young adults: 23 [19–30]	Experiences of transitional care for adolescents with juvenile idiopathic arthritis	Focus Groups	Interpretative Phenomenological Analysis	Transitional care: multi-dimensional coordinated supportive developmentally appropriate age-appropriate
									Transfer from paediatric to adult services Preparation for transfer
Sly, Morgan [22]	2014	Anorexia Nervosa	Emerging Adults	8 patients	25 [18–34]	Experiences of therapeutic alliance during in-patient treatment for anorexia nervosa	Interview	Interpretative Phenomenological Analysis	Alliance as a key experience Active, not passive Taboo talking First impressions count
Stewart, Law [68]	2001	Physical disabilities	Emerging Adults, Parents	21 patients, 12 parents. One service provider also interviewed	23.2 [19–30]	Experiences of transition for young people with physical disabilities	Interview	Editing style of Thematic Analysis	The Context: “Trying to Fit" The Transition Process: “Changes and Cliffs” Needs and Services: “Building a Bridge”
Swift, Hall [69]	2013	Attention Deficit Hyperactivity Disorder	Emerging Adults	10 patients	[17–18.5]	Experieces of transition to adult mental health services	Interview	Thematic Analysis	Clinician qualities and relationship Responsibility of care Nature and severity of problems Expectations of AMHS
Webster and Harrison [71]	2008	Mental Health Disorders	Emerging Adults	20 patients	[18–25]	Experiences of the onset of mental health problems, and initial interactions with the health system	Interview	Grounded Theoretic Analysis	First sign Recognition Understanding Resolution Maze to care model

As all 24 papers that passed the quality appraisal step were qualitative papers or a systematic review of qualitative papers, a meta-aggregation methodology, adapted from that proposed by Lockwood et al. [20], was chosen due to its applicability to a variety of types of papers. In this method, each paper was read, and the findings identified and extracted, along with a unit of data (in this case, a quote from a participant) that supported each finding. Findings that were unsupported by data were not recorded, and where practitioners were also involved as participants in a study, only statements attributable to patients or family members were used to identify findings and associated data. These findings were then collated into groups of similar findings, from which overall themes were synthesised. In several cases, no verbatim quote from the paper could be found that adequately summarised the finding, and so Lockwood’s methodology was modified by allowing the researchers to reword findings slightly to reflect the context of the report in which the finding was identified. For example, the direct finding “Assumptions were often experienced as being over-simplistic, undermining, patronising, and accusational” was extracted from one study, supported by the direct quote “You are rational sometimes, and it did annoy me sometimes that it was ‘it’s the anorexia talking’ and it’s like ‘no it is me!’” [21], while the finding “Relationships can be unbalanced if the provider is too passive” was developed from the “Approximately three quarters of participants talked about past experiences of key nurses with whom they had a relationship which was felt to be unbalanced. Some thought it was in terms of the key nurse being too domineering, […] or indeed too passive”, supported by the direct quote “I was able […] to dominate them, just run the programme and [nurse’s name] was really nice and friendly and all, but couldn’t control it, me.” [22].

## Results

**Figure 2:**
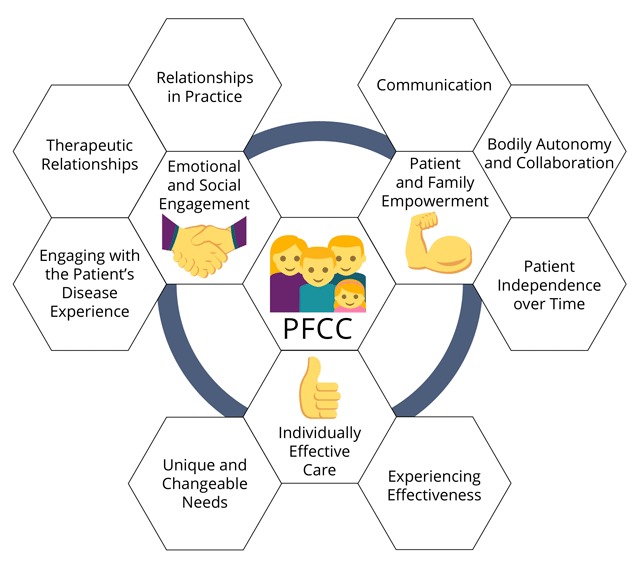
Characteristics and subthemes identified as being part of PFCC.

Emerging adult patients with chronic diseases and their families experienced high-quality PFCC as having three major characteristics: (1) patients and practitioners felt able to engage with each other on an emotional and social level, (2) patients and families felt empowered to be part of the care process, and (3) patients and families experienced care as effective. These characteristics and their relevant subthemes are pictorially depicted in Figure 2, which was developed by the first author, and further discussed below.

### Theme 1: Emotional and Social Engagement

Participants stressed the importance of the practitioner, patient, and family interacting on an emotional and social level, which facilitated information sharing, self-management, and long-term engagement with treatment. They also suggested ways in which practitioners could enhance relationships in practice. A close relationship helped practitioners recognise and treat the patient’s unique experience, rather than focusing on expected disease process and symptoms.

#### Therapeutic Relationships

Strong relationships with practitioners were central to the experience of PFCC, and were both “care capital” that facilitated and enhanced treatment and a reflection of the perceived quality of the care received [22,58]. Communication was enhanced when patients felt comfortable with and trusted their practitioners [22,35,39,43,54,62,67]. The development of trust required time, and was hampered by lack of continuity of practitioners [35,44,58,67].

“They were there for me …. If it was just another program I wouldn’t have honestly cared, I would have just disappeared …. But … they put the time and effort into trying to help me [and] all they ask from me is just to be better.” [52]

Patients and families emphasised their individual strengths and capabilities, particularly their expertise about the disease and its management [43,65,68]. Where practitioners did not recognise and value this expertise, participants felt that they were not being taken seriously [43,52]. However, they also acknowledged that changes in experience over time led to changes in their understanding and ability to advocate for themselves, requiring a flexible approach by practitioners [32,34,44,62].

Participants felt it important that practitioners treat them the same as they would any other person, fostering a sense of normality [21,58,62]. Stereotypes held by practitioners based on the patient’s age or disease left patients feeling like their practitioners did not care about them, disrupting the therapeutic relationship [21,44,58,68].

The provision of emotional support, not just medical support, was an important part of the relationship [21,39,42,58,63], and supported the development of trust [32,43]. Importantly, a lack of emotional support lead to patients “burning out” over time, even when their care was medically appropriate [24,58].

“If I’m sad or feel alone, I would call my social worker for an appointment … we can meet and talk not only about the test results.” [63]

The relationship between the patient and family was also described as a source of support for young people [52,62]. Treatment programmes that did not acknowledge this relationship left patients feeling further isolated and frustrated [21]. Young people trusted family members, particularly parents, to have their best interests at heart [43]. Family members were affected by the patient’s symptoms [62] and needed resources and support [43]. When appropriately supported, family members helped facilitate treatment success by supporting shared decision-making and patient engagement with the health care process [34,54,62,71].

“My mom comes with me every time … I actually like her support … Having my mom come makes it feel less of a struggle.” [52]

#### Relationships in Practice

Participants highly valued practitioners who demonstrated a keen and ongoing interest in their lives and wellbeing [22,43,54,62]. Listening attentively to understand patient and family perspectives was strongly emphasised as valuable, facilitating communication and enhancing treatment effectiveness [22,32,35,43,44,49,54,58,69]. Understanding the patient’s experience of disease severity could also help to ensure that therapeutic messages were not seen as catastrophizing or exaggerated [55].

Conversely, practitioners who did not listen to or consider the concerns and opinions of patients and families could discourage them from attending appointments [22,55,62]. Particularly effective were practitioners who created a relaxed and safe atmosphere, in which both parties could share personal stories, concerns, and experiences [21,22,49,52].

“I’d look forward to our [weekly] sessions […] I knew I could keep going because soon we’d have key work and talk it through.” [22]

Practitioners who made time for patients and families were very positively regarded [24,30,52,69], particularly when available outside scheduled appointment times or away from the office [49,52]. Participants recognised that practitioner time is scarce and did not want to waste that time, and so time spent was highly valued as a result [24,49,62].

“They would make the effort, and I like that. Instead of waiting for me to come to them, they would come to me, call me, ask me what’s wrong you know … So now I like to come, and I look forward to talking with them.” [52]

#### Engaging with the Patient’s Disease Experience

The experience of chronic disease was complex, with effects across many areas of life. The effect of disease on social networks was especially important, and effective treatment supported the maintenance of existing social relationships and the development of new ones [43,68]. Comparing their own experiences against those of their healthy peers could leave young patients feeling abnormal [35,63], further isolating them from their peers and leaving them feeling lonely [54,62], or as if their lives had become stagnant [21,62].

“But I was very aware that they were getting on with their lives, erm, they were doing their ‘A’ levels, they were gonna be going off to university at the end of the year, and that was really hard for me cos I had fears of everybody going … and I’d never catch up … it meant that I sort of stayed stuck.” [21]

This sense of abnormality and loneliness induced fear of relapse [54] and grief [35]. This could lead to denial of the disease and its effects [55,62], to re-establish a personal sense of being “normal” [55]. Practitioners’ failures to engage with these emotions could get in the way of the patient engaging with them [24,58]. Conversely, where strong disease-related emotions kept the young person from engaging with treatment, more directive treatment approaches could allow them to become more comfortable and self-sufficient [21,24].

Patients stressed the importance of practitioners considering the complexities of their lives [52,54], particularly the ways in which non-medical factors like housing needs [42,58], employment [62], and lack of a daily routine [39] could interfere with their ability to participate in treatment. To this end, patients valued providers who recognised the negative impacts that treatment could have on everyday life and assisted them to minimise them [39,54].

“Right now, I do need professional help … and the thing that’s stopping me is basically time. I was going to school full-time, then I have to come home and take care of my daughter. So it’s just a battle between when do I take the time to do it?” [54]

Young people developed expertise in their own care that sometimes superseded that of the medical team [55,58]. Peers who had the same health conditions, and therefore similar expertise, could be valuable emotional supports [21,24,42,58,62] or mentors [58,67], and could act as guides in ways that practitioners could not.

“If they had somebody that they could talk to that’s their own age that is going through some of the issues that they’re going through, you know, I think that’d be really powerful.” [42]

### Theme 2: Patient and Family Empowerment

Participants expected practitioners to enable and empower them to engage in care collaboratively, rather than be passive recipients of medical expertise. The quality of communication was the primary facilitator of collaboration, with poor communication by practitioners disempowering patients and families. The kind and intensity of collaboration and communication required changed as young adults aged and gained increased autonomy and independence.

#### Bodily Autonomy and Collaboration

Patients repeatedly emphasised the importance of practitioners recognising ownership of their bodies [67] and power in their own lives [43]. Information, both medical and service-based, facilitated a sense of control in patients. In particular, the rights to “know what’s going on with your body” [58], to initiate help-seeking [43], and to determine to whom medical information was disclosed [63] were deeply important to patients. Relevant information, communicated clearly, and at an appropriate level of complexity, was highly valued [35,43], as it helped patients and families better understand and predict disease [62]. Patients could also use this information, given the chance by their practitioners [43], to develop effective self-management strategies [43,62].

Even with full and frank disclosure of medical information, patients and families were often unaware of services that could be beneficial to them [30,54], in some cases learning about health services via serendipitous encounters with other professionals [54]. Practitioners were expected to proactively fill these information gaps [62], often because patients and families “don’t know what they don’t know” [30].

“The services are there. Sometimes you have to ask specifically. Like they don’t just sort of say ‘well these are the services that are out there for you.’ You have to say ‘I want this’. And then they’ll tell.” [30]

Participants wanted to collaborate with practitioners [21]: discuss their options and the potential benefits of those options [43,58], ask questions [62], take time to consider the information [32], and then make decisions for themselves [21,24,43,62]. Care that was collaboratively determined was valued [21], and the resulting feeling of empowerment helped patients feel more in control of their own disease [43] and improved their motivation and engagement with treatment [22,24,34,52].

#### Communication

Families often had trouble understanding practitioners, and felt that being clearly understood was part of the practitioners’ role [30]. In particular, staff making decisions without involving the patient and family left participants feeling confused and frustrated [21,62], disengaged with treatment [52,58] and powerless [21]. In addition, by not proactively informing and including patients, practitioners excluded patients from forward planning and decision-making, leading some to believe that none had been done at all [58,62,67].

“I don’t think my doctor thought about it. There were a lot of things that I didn’t know or didn’t think about, and I kind of went through things blind.” [58]

Difficulties communicating were compounded by fear of speaking to practitioners. In some cases, patients did not know that they could assert themselves [32], or feared that by asserting themselves they would assume sole responsibility for care, losing the support of health staff [62]. Where patients were able to assume independence gradually, they were more confident and better able to self-advocate [32,62], although failure to assert or manage newfound power quickly eroded confidence [21,67].

#### Patient Independence over Time

As the primary drivers of their children’s care over the long term, parents felt insight into their children and their condition, with mothers feeling that they could recognise symptoms and concerns before clinicians did [34,62]. As a result, parents felt a right to be directly involved in their young adult children’s care [65], and a responsibility to ensure that they were protected from substandard care [65,67], unscrupulous providers [64], and immature decision-making [65].

However, parents wanted their children to develop independence and self-management, and encouraged them to see providers alone and be active in appointments [64,65,67]. Parents recognised incompleteness of their knowledge about their children, and trusted their adult children and practitioners to share information and work in the child’s best interests [65].

“The whole role for me of being a parent is to get them to that independent stage where they can think for themselves and do for themselves and be able to start to relate to other people in all aspects of their life.” [65]

Parental intention was not sufficient for young people to achieve independence, with practitioner support required. Young people wanted to be able to see their practitioners alone [44,67], and gain access to information [67], but had trouble telling their parents this [67], especially in situations where their parents had “trouble letting go” [24,58]. In extreme cases, parental involvement was a barrier to treatment, dissuading patients from treatment [52,71]. Clinicians could facilitate patient involvement in consultations [35], although this could be as simple as addressing them directly, rather than the parents [67].

### Theme 3: Individually Effective Care

Participants defined effective care as not just that which improved medical outcomes, but as care in which the individualised needs of patients and families were addressed in a way that they felt worked for them. Patients needed to easily access experienced and knowledgeable professionals, as care delivered by practitioners who were not available or not perceived as skilful was not felt to be effective.

#### Unique and Changing Needs

Patients’ needs were unique [35,42], and dependent on the life and goals of the person themselves [52], which required flexibility on the part of the practitioner [35]. Patients and families wanted to discuss the approach to care [34] and treatment methods [62] so that decisions could be tailored to their particular circumstances.

“If you leave it up to the individual to pick goals or things that are essentially problems for them and they are working towards that, they can see the benefit of their improvements, and obviously they’re a lot more satisfied with that.” [34]

In particular, medication as a first resort treatment was a warning sign that the care team did not truly understand what the problems were [49]. Young people had complex attitudes towards medication [49,62], although they were more likely to accept it if they chose it for themselves [52].

Young people’s needs change over time [39], and this was particularly visible during transition from paediatric to adult services. Participants felt that services were withheld as young people got older [54,68] without regard for their needs, and that services that were provided were tailored towards younger children [67]. A patient’s individual needs and capabilities were more important for transition readiness than age [24,44,62,67,69].

“… it’s not about the age. I don’t believe anything is about the age. He (Psychiatrist) looked at it (referral) with other people and said you know where do you think, who would be best for her?… I think that would be better for people to do that rather than put them in a category because of their age because I don’t think that’s fair. We need services based on our needs not our bloody age.” [44]

Addressing those needs that mattered most to the patient and family led to more obvious benefits. Patients preferred treatment that they felt provided obvious benefits [34,42,49], and engaged more with services that they felt helped them [52,54]. Services that they did not feel helped were discouraging [49], and they avoided services or treatments without obvious personal benefits [52,54].

#### Experiencing Effectiveness

To experience care as effective, young people needed to feel that they could access it when they needed to, and that this access would continue into the future. Flexibility in appointment times [39] and services that were made available outside standard appointment structures [69] reduced anxiety around short-term access. Long wait times, a lack of insurance, and reliance on public transportation [54] were all barriers to access. Young people were particularly concerned that their access would diminish over time as they moved away from paediatric services [62,68,69].

Patients and families wanted to deal with professionals who demonstrated knowledge and skill [49], were experienced in working with young people [30,35], and had in-depth understanding of the health condition [44,62]. They acknowledged that practitioners (in particular General Practitioners) may not have these skills [43] and would rather be referred to a specialist than seen by someone without appropriate training and expertise [39]. Unprofessional conduct [49] and inconsistent information [62] made care feel ineffective, which reduced trust, especially among parents [65].

## Discussion

These characteristics of PFCC reflect the views of a wide range of young people living with chronic disease and their families. While they are necessarily interdependent, they reflect the broad diversity of what young people and their families want from chronic disease management.

The development of a Therapeutic Relationship, driven by the practitioner’s recognition of the patient’s unique experience, is a powerful facilitator of communication and trust. By recognising relationships as “care capital” with inherent value to the health care process, practitioners can prioritise the development of these relationships, refocusing therapeutic interactions towards the person and their experience, rather than the disease.

Empowerment of Patients and Families was a feature of all stages of disease management, from the initial recognition of patients’ personal autonomy by including them in decision-making to the gradual transition of control to patients from their parents and other caregivers over time. By encouraging collaboration through welcoming and encouraging active communication (supported by strong social and interpersonal relationships as discussed above), practitioners can enhance communication. This allows patients to establish themselves as part of the health care team rather than passive subjects of medical intervention.

Once these foundations of strong emotional and social engagement and patient and family empowerment were laid down, patients and practitioners could work towards addressing patients’ individual needs. Recognition of the unique and changing nature of the needs of patients and their families places practitioners in a powerful position to facilitate fulfilling those needs. By assisting patients to directly and meaningfully experience the achievement of goals, practitioners can demonstrate effectiveness, encouraging engagement on an ongoing basis and helping to ward off “burnout”. This experience of success also may help foster a sense of hope for the future, a component of patient-centred care particular to chronic disease settings [13].

These components are quite similar to extant models of patient-centred care in the published literature. In particular, engagement between patient and family and practitioner is reflected in the themes of “patient-as-person”, “doctor-as-person”, and “the therapeutic alliance” presented in the model of PCC developed by Mead and Bower [6]. Both models highlight the importance of an honest and open relationship in which both parties interpersonally influence each other.

This may be contrasted with the model of PCC developed by Kitson et al. [72], where there is much less emphasis on the practitioner as an emotional and social actor in the health care exchange. While their model highlights cooperation between practitioners and patients, it does not address the facilitators of this cooperation, such as continuity of care. The ongoing nature of the patient/practitioner partnership, was identified by Hudon et al. as being more important in chronic disease settings than acute settings due to the duration of care [13], which may explain why this is not obvious in Kitson’s study of acute care settings [72].

Similarly, the importance of patient and family empowerment reflects Mead’s [6] focus on “sharing power and responsibility” and Kitson’s [72] concept of the “patient participating as a respected and autonomous individual”, as well as their recognition of the personal expertise of the patient and the importance of open communication of knowledge. Again, the direct empowerment of the patient to deliver care and support their own health is not as strong in Kitson’s [72] model, which was also identified by Hudon et al. [13] as more prominent in chronic disease settings than acute settings.

The sub-theme Bodily Autonomy and Collaboration, focusing on young people’s sense of their right to make their own decisions and lead their own care, is similar to the importance of “family choice” identified by Epley et al. in their model of family-centered care (FCC) [73]. Their recognition of individualised services as important parts of FCC was mirrored by young adults in the literature, with services only being experienced as effective if they addressed patients’ unique needs. Epley et al. also recognised the family-professional relationship, similar to the current results. However, their presentation of the family as the unit of attention reveals an interesting tension: the role of parents in the health care process is not clear in our results, with parents acknowledging that they had to work to step back and allow their children additional agency and autonomy over time.

In contrast, there are significant differences between the present results and the Neurodevelopmental Clinical Research Unit framework for FCC developed by Rosenbaum et al. [8]. This model highlights the parents as the unit of agency within the family, rather than the family as a support to a child able to make decisions. Having been developed in a child rehabilitation context, this model addresses the experiences and needs of children, rather than those of young adults. Children have yet to develop the personal autonomy and capabilities that are characteristic of what Arnett called “emerging adulthood”, and so models of family-centred care that focus on young children may be unable to incorporate these capabilities into their structure.

### Limitations

Papers identified in this systematic review largely focused on the views of emerging adult patients to the exclusion of their family members: only seven incorporated the views of parents, only one included siblings, and none incorporated other family members such as grandparents, partners, or children. Where parents were included as participants, their role in facilitating their children’s eventual independence and success was emphasised, and this seems to be an important part of patient- and family-centred care in this population.

The sample also focused on experiences of transition from paediatric to adult services. This is a time of significant change for young people living with chronic health conditions, and the findings of this review suggest that young people and their families desire stability and continuity in their interactions with practitioners. As young people transition between services and service providers, they may focus on this lack of stability, de-emphasising other components of PFCC in the interest of addressing the primary threat to their sense of safety.

The present review of qualitative studies is, necessarily, two steps removed from the experiences of the young people who responded to the identified studies. While the research team have attempted to focus on the words of participants in the synthesis process, and highlight their voices in the results, identified findings have been shaped by the decisions of the individual researchers who conducted the component studies, and further interpreted by the current research team. This repeated interpretation by health researchers may privilege the development of concepts already familiar to health researchers – for example, shared decision-making, patient involvement, and the therapeutic relationship [74]. As a result, reviews like this should not be assumed to reflect the wishes and needs of young adult patients in every care setting. Rather, directly engaging with patients and working with them to co-design services and service improvements is vital [75].

## Conclusion

These findings support the applicability of several existing approaches to PFCC among young adults living with chronic health conditions and their family members, which has not previously been established in the literature. They also highlight several issues with applying existing models of family-centred care developed in child health to this population, which may be important for child health practitioners working with patients as they move through young adulthood. The findings of this study highlight actions that health care practitioners could take to encourage PFCC in their everyday practice, as seen through the eyes of young people. In this way, they complement the more theoretical framework put forward by Stewart et al. that suggests a way of conceptualising health care and disease to enable patient-centred care [3].

The present results suggest three immediate measures of patient- and family-centredness that may be useful for clinicians as part of reflective practice: (1) Did I engage emotionally with my patient and their family on an honest level; (2) Did I empower the patient and their family to participate in decision-making and health-care delivery; (3) Did I focus care on the goals of the patient and family as they see them? In this way, practitioners may be more able to assess their own practice to better ensure that they are delivering care to their young adult patients in a patient- and family-centred manner.

Further research in this area should incorporate the experiences and opinions of parents and other family members to be sure that this important facilitation role is being enhanced as control of the clinical process passes from them to their young adult child. Further investigation in settings where transition is not a feature would be helpful in elucidating additional features of PFCC that may emerge when existing practitioner relationships are not under threat.
